# Structural and functional characterization of TesB from *Yersinia pestis* reveals a unique octameric arrangement of hotdog domains

**DOI:** 10.1107/S1399004715002527

**Published:** 2015-03-27

**Authors:** C. M. D. Swarbrick, M. A. Perugini, N. Cowieson, J. K. Forwood

**Affiliations:** a School of Biomedical Sciences, Charles Sturt University, BLD 289, Wagga Wagga, NSW 2678, Australia; b Department of Biochemistry, La Trobe Institute for Molecular Science, La Trobe University, Bundoora, VIC 3086, Australia; c Department of Biochemistry and Molecular Biology, Bio21 Molecular Science and Biotechnology Institute, Parkville, VIC 3010, Australia; d Australian Synchrotron, Clayton, VIC 3168, Australia

**Keywords:** TesB, acyl-CoA thioesterases, *Yersinia pestis*

## Abstract

Acyl-CoA thioesterases catalyse the hydrolysis of the thioester bonds present within a wide range of acyl-CoA substrates, releasing free CoASH and the corresponding fatty-acyl conjugate. The TesB-type thioesterases are members of the TE4 thioesterase family, one of 25 thioesterase enzyme families characterized to date, and contain two fused hotdog domains in both prokaryote and eukaryote homologues. Only two structures have been elucidated within this enzyme family, and much of the current understanding of the TesB thioesterases has been based on the *Escherichia coli* structure. *Yersinia pestis*, a highly virulent bacterium, encodes only one TesB-type thioesterase in its genome; here, the structural and functional characterization of this enzyme are reported, revealing unique elements both within the protomer and quaternary arrangements of the hotdog domains which have not been reported previously in any thioesterase family. The quaternary structure, confirmed using a range of structural and biophysical techniques including crystallography, small-angle X-ray scattering, analytical ultracentrifugation and size-exclusion chromatography, exhibits a unique octameric arrangement of hotdog domains. Interestingly, the same biological unit appears to be present in both TesB structures solved to date, and is likely to be a conserved and distinguishing feature of TesB-type thioesterases. Analysis of the *Y. pestis* TesB thioesterase activity revealed a strong preference for octanoyl-CoA and this is supported by structural analysis of the active site. Overall, the results provide novel insights into the structure of TesB thioesterases which are likely to be conserved and distinguishing features of the TE4 thioesterase family.

## Abstract 2

Structural and functional characterization of the TesB thioesterase from *Y. pestis* reveals unique elements within the protomer and quaternary arrangements of hotdog domains, presenting distinguishing features of this enzyme family.

## 1. Introduction   

Acyl-CoA thioesterases (Acots) perform a wide range of cellular functions through their catalysis of activated fatty acyl-CoA molecules to their respective fatty-acyl and CoA products. They are ubiquitously expressed throughout evolution and play important regulatory roles ranging from inflammation (Forwood *et al.*, 2007 ▶; Swarbrick *et al.*, 2011 ▶), lipid biosynthesis, signal transduction and allosteric regulation of enzymes (Kirkby *et al.*, 2010 ▶) in eukaryotes to fatty-acid elongation, regulation of membrane biosynthesis and negative regulation of genes involved in fatty-acid and phospholipid biosynthesis in prokaryotes (Dillon & Bateman, 2004 ▶). Owing to the diverse nature of these enzymes, the thioesterase superfamily has been classified into 25 families based on sequence homology, substrate activity and tertiary structure (Cantu *et al.*, 2010 ▶).

The TesB-type thioesterases are unique within the thio­esterase superfamily, containing a double-hotdog fold in both prokaryotes and eukaryotes, a feature which is observed in only one other thioesterase class (TE6). To date, the structural features of only two active TesB-type thioesterases have been resolved, those from *Escherichia coli* and *Mycobacterium marinum*; however, a further two structures have been solved and annotated as TesB thioesterases from *M. avium* (PDB entries 3rd7 and 4r9z; Seattle Structural Genomics Center for Infectious Disease, unpublished work; C. M. D. Swarbrick & J. K. Forwood, unpublished work) but do not contain active sites characteristic of thioesterases. Thus, much of the current understanding regarding this class of thioesterases been built on the *E. coli* structure, with the other TesB structure, that from *M. marinum*, which was deposited in the Protein Data Bank by the Seattle Structural Genomics Center for Infectious Disease, remaining to be published. The structure of the TesB thioesterase from *E. coli*, published by Li *et al.* (2000 ▶), was shown to form a double-hotdog fold, with each fold exhibiting an antiparallel β-sheet forming the ‘bun’ of the hotdog surrounding a central α-helix ‘sausage’. These two hotdog domains associate together with the two central α-helices aligning together and the two β-sheets associating through strands 3 and 9 to form an extended β-sheet. In other thioesterase families (TE2, TE6 and TE7), these hotdog folds self-associate into higher order configurations depending on the thioesterase class, ranging from dimers (Dias *et al.*, 2010 ▶) and tetramers (back to back or face to face; Tilton *et al.*, 2004 ▶) to hexamers (trimer of dimers configuration; Forwood *et al.*, 2007 ▶; Pidugu *et al.*, 2009 ▶). Based on the structure of the *E. coli* TesB structure, the TesB enzymes are believed to function as a double-hotdog protomer (Li *et al.*, 2000 ▶; Pidugu *et al.*, 2009 ▶). Since our knowledge of TesB thioesterases is largely based on one thioesterase, we set out to structurally and functionally characterize TesB from *Yersinia pestis* in order to better understand this class of enzyme and its possible role in this pathogenic organism.

*Y. pestis*, the causative agent of bubonic plague, is a highly virulent bacterial pathogen capable of rapid dissemination throughout the body. Untreated infections rapidly develop into high-density septicaemia that is generally fatal. If the bacillus reaches the lungs, transmission from human to human may occur through cough droplets containing the plague bacteria, and cause pneumonic plague, which carries a fatality rate of 100% within 1–3 d of onset of symptoms if not treated (Kool, 2005 ▶). Historically, bubonic plague has been responsible for numerous epidemics, with one epidemic in the Middle Ages responsible for killing one quarter of the European population (Cabanel *et al.*, 2013 ▶; Tourdjman *et al.*, 2012 ▶). Presently, infections still occur in the United States, Africa and Asia, and the occurrence of antibiotic resistance has been reported (Galimand *et al.*, 2006 ▶), but perhaps the greatest present-day threat of outbreak is through bioterrorism, where *Y. pestis* is listed as a category A agent on the CDC Bio­terrorism threat list, and it is expected that the majority of the resulting cases of plague would be pneumonic (Inglesby *et al.*, 2000 ▶; http://www.bt.cdc.gov/agent/agentlist-category.asp).

The TesB acyl-CoA thioesterase from *Y. pestis* (*Yp*TesB) is a 288-amino-acid protein and is a homologue of the human HIV Nef-binding protein ACOT8, sharing ∼40% amino-acid sequence identity. Here, we describe the structure and function of the acyl-CoA thioesterase TesB and provide comparisons with other thioesterases within this family. The high-resolution crystal structure exhibits a unique octameric arrangement of hotdog domains that has not been described in any other thioesterase or protein. We confirm the quaternary structure using small-angle X-ray scattering, size-exclusion chromatography and analytical ultracentrifugation, and assess the substrate specificity across a range of short-, medium- and long-chain fatty acyl-CoA molecules. Specificity is exhibited for medium-chain fatty acyl-CoAs, and this is supported by analysis of the active-site tunnel. Overall, our results provide novel insights into the TesB thioesterases both at the protomer and the quaternary level, which are likely to be both conserved and defining features of the TE4 thio­esterases.

## 2. Materials and methods   

### 2.1. Protein expression and purification   

The gene encoding TesB from *Y. pestis* was amplified by PCR and cloned into the expression vector pMCSG21. The protein was recombinantly expressed in *E. coli* BL21 (DE3) pLysS cells as a His-tagged fusion protein and induced by the addition of 1 m*M* IPTG at an OD_600_ of 0.6. The protein was purified by Ni^2+^-affinity chromatography and elution fractions were pooled and incubated overnight with TEV protease (30 µg ml^−1^) to remove the affinity tag. The protein was further purified using size-exclusion chromatography in 50 m*M* Tris pH 8.0, 125 m*M* NaCl, and a single peak was collected, concentrated to 20 mg ml^−1^, aliquoted and stored at −80°C.

### 2.2. Crystallization and structure determination   

Crystallization was performed using the hanging-drop vapour-diffusion method as per Swarbrick *et al.* (2013 ▶). Crystals were obtained by mixing 1.5 µl protein solution at 20 mg ml^−1^ protein with 1.5 µl reservoir solution consisting of 20% PEG 3350, 235 m*M* sodium malonate pH 7.0, 1 m*M* CoA; crystals containing CoA in the structure were obtained using a reservoir solution consisting of 22% PEG 3350, 200 m*M* sodium malonate pH 7.0, 1 m*M* octanoyl-CoA. Protein crystals were cryoprotected by briefly soaking the crystals in reservoir solution containing 15% glycerol. Diffraction data were collected on the MX2 beamline at the Australian synchrotron and the images were indexed and integrated in *MOSFLM* (Leslie & Powell, 2007 ▶) and scaled using *AIMLESS* (Evans, 2011 ▶). Phases were determined by molecular replacement in *Phaser* (McCoy *et al.*, 2007 ▶) using chain *A* of PDB entry 1c8u (Li *et al.*, 2000 ▶) as a model. Refinement and model rebuilding were undertaken in *Coot* (Emsley *et al.*, 2010 ▶) and *REFMAC*5.8 (Murshudov *et al.*, 2011 ▶). The structures of apo *Yp*TesB and CoA-bound *Yp*TesB have been deposited in the RCSB Protein Data Bank as entries 4qfw and 4r4u, respectively.

### 2.3. Mutagenesis   

Mutagenesis was performed using the QuikChange site-directed mutagenesis kit (Stratagene). The reaction mixture consisted of 40 µl distilled H_2_O, 5 µl reaction buffer, 1 µl dNTP mix, 1 µl *PfuUltra* High-Fidelity DNA Polymerase, 50 ng plasmid and 125 ng each of the forward and reverse primers. The reaction mixture was heated to 95°C for 30 s followed by 16 cycles of 95°C for 30 s, 55°C for 1 min and 68°C for 3.5 min. The mixture was then incubated at 37°C for 1 h with DpnI restriction enzyme to digest the parental DNA prior to transformation into XL1-Blue supercompetent cells. The fidelity of the clones was confirmed by DNA sequencing.

### 2.4. SAXS data collection   

Small-angle X-ray scattering (SAXS) data were collected on the SAXS/WAXS beamline at the Australian Synchrotron using a Pilatus 1M detector. Samples of *Yp*TesB were prepared by serial dilutions to 5, 2.5, 1.25, 0.6 and 0.3 mg ml^−1^ and elution buffer was used for subtraction. 50 µl of sample was drawn through a 1.5 mm quartz capillary and exposed to the X-ray beam. The scattering data were collected from *q* = 0.009 to 0.541 Å^−1^ and were reduced to remove background buffer and capillary noise/scattering.

Detector images for each concentration were averaged using *scatterBrain* (written and provided by the Australian Synchrotron; available at http://www.synchrotron.org.au/) to generate a number of SAXS data sets for subsequent analysis using *ATSAS* v.2.4.3. *PRIMUS* (Konarev *et al.*, 2003 ▶) was used to subtract background scattering from data files and Guinier fits and *P*(*r*) distribution plots were generated using *GNOM*. *CRYSOL* (Svergun *et al.*, 1995 ▶) was used to generate theoretical curves and to compare the scattering data with the crystal structure.

### 2.5. Analytical ultracentrifugation data collection   

Sedimentation-velocity experiments were conducted in a Beckman model XL-A analytical ultracentrifuge at 20°C. TesB solubilized in 50 m*M* Tris, 125 m*M* NaCl pH 8.0 was analyzed at an initial concentration of 2.4 mg ml^−1^. 380 µl of sample and 400 µl of reference solution were loaded into conventional double-sector quartz cells, mounted in a Beckman four-hole An-60 Ti rotor and centrifuged at a rotor speed of 40 000 rev min^−1^. Data were collected at a single wavelength (296 nm) in continuous mode using a step size of 0.003 cm without averaging. An estimate of the partial specific volume (0.732 ml g^−1^ at 20°C) and shape factors assuming prolate, oblate or cylinder models were computed using *SEDNTERP* (Laue *et al.*, 1992 ▶). Sedimentation-velocity data at multiple time points were fitted to a continuous sedimentation coefficient [*c*(*s*)] distribution and a continuous mass [*c*(*M*)] distribution model (Perugini *et al.*, 2000 ▶; Schuck, 2000 ▶, 2002 ▶) using *SEDFIT* (Schuck, 2000 ▶).

### 2.6. Enzyme assays   

Thioesterase activity was measured by detection of the sulfhydryl group released as a product of the reaction as described previously (Yamada *et al.*, 1999 ▶). The reaction mixture consisted of 100 m*M* phosphate buffer pH 8.0, 0.1 m*M* 5,5′-dithiobis-(2-nitrobenzoic acid) (DTNB) and the enzyme (0.33 µg ml^−1^) in a final volume of 100 µl. The absorbance at 412 nm was measured for 20 min and the activity (expressed as moles of acyl-CoA hydrolysed per minute per milligram) was calculated using ∊_412_ = 13 600 *M* ^−1^ cm^−1^. Substrates screened included acetyl-CoA (C2), malonyl-CoA (C3), butyryl-CoA (C4), hexanoyl-CoA (C6), octanoyl-CoA (C8), decanoyl-CoA (C10), lauroyl-CoA (C12), myristoyl-CoA (C14), palmitoyl-CoA (C16), stearoyl-CoA (C18) and arachidoyl-CoA (C20) sourced from Sigma–Aldrich.

## 3. Results and discussion   

### 3.1. Crystallographic structure determination of apo TesB from *Y. pestis*   

**Figure 1:**
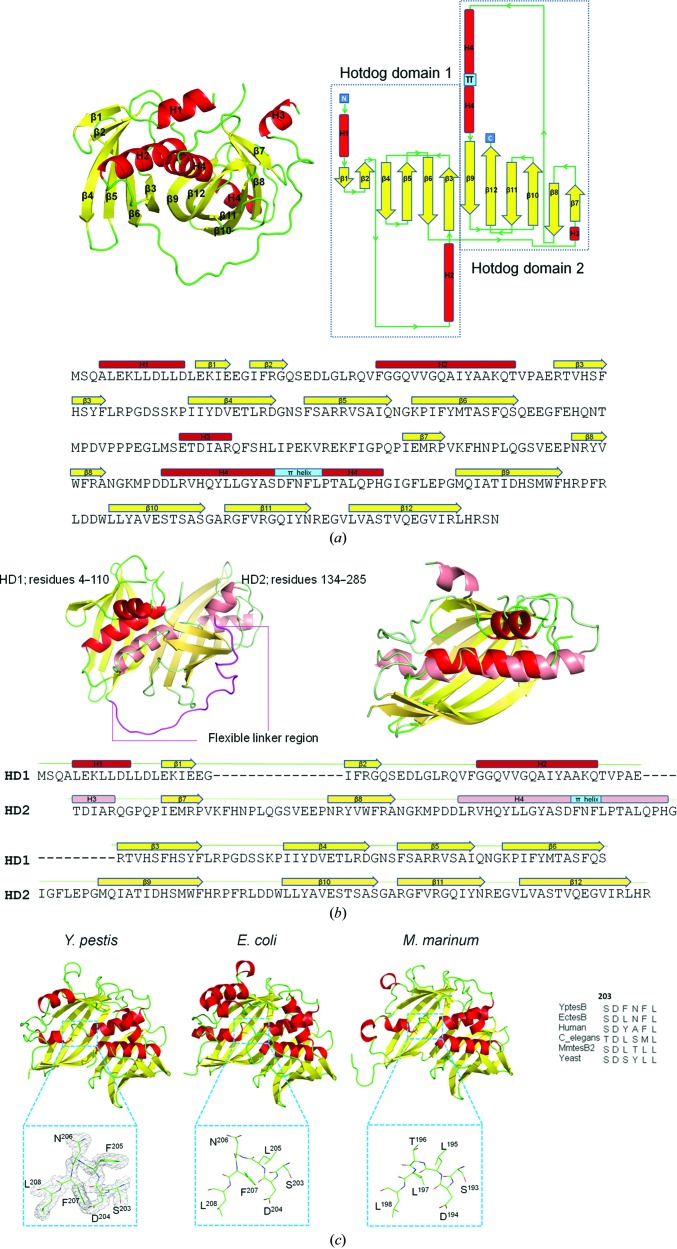
The primary, secondary and tertiary structure of TesB with α-helices coloured red, β-strands yellow and loops green. (*a*) The double hotdog-domain structure of *Yp*TesB. (*b*) The two hotdog domains of *Yp*TesB, with a superposition of the domains (r.m.s.d. of 0.33 Å) and sequence alignment, and (*c*) a comparison of three TesB structures from *Y. pestis* (PDB entry 4qfw), *E. coli* (PDB entry 1c8u) and *M. marinum* (PDB entry 3u0a), revealing a conserved β-bulge through the HD2 α-helix.

Crystals of *Y. pestis* TesB (*Yp*TesB) diffracting to 2 Å resolution and indexed in space group *P*2_1_ (see Table 1 ▶ for full data-collection and refinement statistics) contained four *Yp*TesB monomers in the asymmetric unit. Each monomer was structurally equivalent, with an r.m.s.d. of less than 0.4 Å between any two chains. Clear, contiguous density enabled residues Ala4–His285 to be modelled for each apo *Yp*TesB protomer, with the exception of two flexible loops spanning residues 28–32 and 139–154 (Fig. 1 ▶). These regions could be modelled with more certainty in the CoA-bound *Yp*TesB structure (see below) owing to interactions with the cofactor that stabilized these regions.

**Figure 2:**
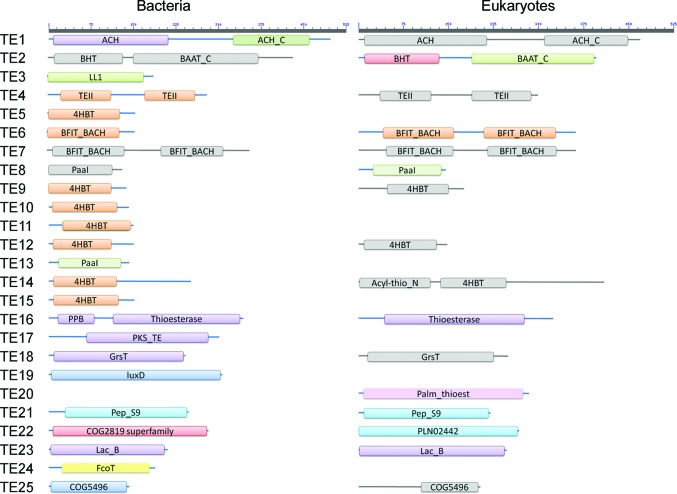
Domain architecture of thioesterase families as presented in the ThYme database (http://www.enzyme.cbirc.iastate.edu/). Abbreviations: ACH, acetyl-CoA hydrolase; ACH_C, acetyl-CoA hydrolase C superfamily; BHT, bile hydrolase transferase; LL1, lysophospholipase L1-like; TEII, thioesterase II; PaaI, phenylacetic acid thioesterase; PPB, phosphopantetheine-binding domain; PKS_TE, polyketide synthase thioesterase; GrsT, gramicidin S biosynthesis thioesterase; luxD, lux-specific myristoyl-ACP thioesterase; Pep_S9, peptidase_S9 superfamily; Lac_B, lactamase_B superfamily. Domains presented in grey have no solved structures and are therefore theoretical.

The TesB monomer is comprised of two hotdog domains arranged in a double-hotdog fold similar to TE4 and TE6 thioesterase family members (Fig. 2 ▶; Cantu *et al.*, 2010 ▶; Forwood *et al.*, 2007 ▶). Each hotdog domain consists of a central α-helix surrounded by a six-stranded antiparallel β-sheet (Fig. 1 ▶), with β-strands arranged sequentially with the exception of β-strand 3 of each domain, which plays a role in dimerization (described below). Despite only 39% sequence identity between the two hotdog domains within the protomer, the hotdog domains are structurally conserved, with an r.m.s.d. (McNicholas *et al.*, 2011 ▶) of 0.33 Å over 78 C^α^ atoms (Fig. 1 ▶ *b*). These domains are linked in the protomer through a long flexible linker that spans residues 111–133 and connects the C-terminus of hotdog domain 1 (HD1; residues 1–110) to the N-terminus of HD2 (residues 134–288) (Fig. 1 ▶ *b*). The domains associate through β-strand 3 of each HD domain (or β-strands 3 and 9 in the protomer; corresponding to residues 56–65 and 224–236, respectively) and two α-helices (helices 2 and 4; residues 35–50 and 191–216, respectively), with an interface area of 1044 Å^2^. This interface is conserved in both *E. coli* TesB (*Ec*Tes; with an interface area of 1250 Å^2^) and *M. marinum* TesB2 (*Mm*TesB2; interface area of 1094 Å^2^), with each interface formed through β-strand 3 and α-helix 2 of each hotdog domain. Residues and interactions that mediate association between hotdog domains within the protomer include Phe64–Ile229, Ser62–His231, Gly37–Asp204, Phe60–Met233, Glu27–Tyr201, Gln49–Tyr197, Val57–Gln196, His58–Phe235, Phe60–Met233 and Ser62–His231.

### 3.2. The TesB tertiary structure has been highly conserved throughout evolution   

Structural conservation of this protein fold was assessed using *DALI* (Holm & Rosenström, 2010 ▶), revealing two structures with an r.m.s.d. of less than 2 Å. A comparative analysis of these structures, *Ec*TesB (PDB entry 1c8u; r.m.s.d. 0.6 Å; 80% sequence identity; Li *et al.*, 2000 ▶) and *Mm*TesB2 (PDB entry 3u0a; r.m.s.d. 1.4 Å; 44% sequence identity; Seattle Structural Genomics Center for Infectious Disease, unpublished work), revealed a conserved secondary-structure topology of α–β–β–α–β–β–β–β–α–β–β–α–β–β–β–β, with each domain represented by the sequence α–β–β–α–β–β–β–β. Notably, this is in contrast to the secondary structure assigned to this thioesterase class by Cantu *et al.* (2010 ▶): α–β–β–β–β–β–β–α–β–β–β–β. The double-hotdog protomer of TesB is also structurally similar to eukaryotic members of the TE6 thio­esterase family (Fig. 2 ▶); however, a distinguishing feature appears to be that TE6 hotdog domains contain two additional α-helices located at the C-terminus of each HD domain.

**Figure 3:**
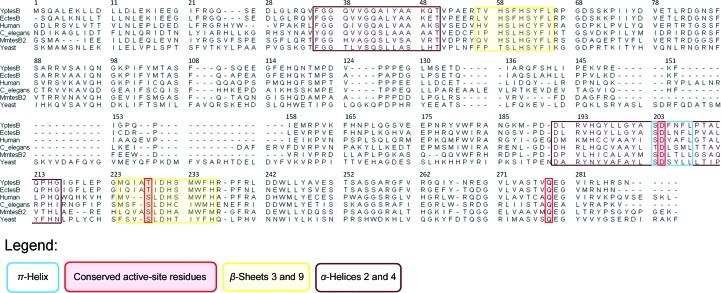
The primary sequence of the π-helix (in blue) is conserved throughout these proteins and spans Asp204 of the active site.

Interestingly, a π-helix was shown to interrupt the central α-helix of HD2, and this is structurally conserved in the other TesB structures (Fig. 1 ▶). In the *Yp*TesB structure, this π-helix is comprised of six residues spanning SDFNFL^208^, which are conserved amongst other TesB sequences with a consensus sequence SD*XX*FL (Fig. 3 ▶). This π-helix harbours the active-site residue Asp204 (Fig. 3 ▶) and is thus consistent with recent reports that π-helices map to functionally important regions of proteins (Cooley *et al.*, 2010 ▶). Significantly, the π-helix identified across TesB structures is not present in other thio­esterases and thus may represent an important structural feature in differentiating thioesterase family members (Marfori *et al.*, 2011 ▶; Willis *et al.*, 2008 ▶).

### 3.3. TesB exhibits a unique quaternary arrangement comprised of an octamer of hotdog domains   

**Figure 4:**
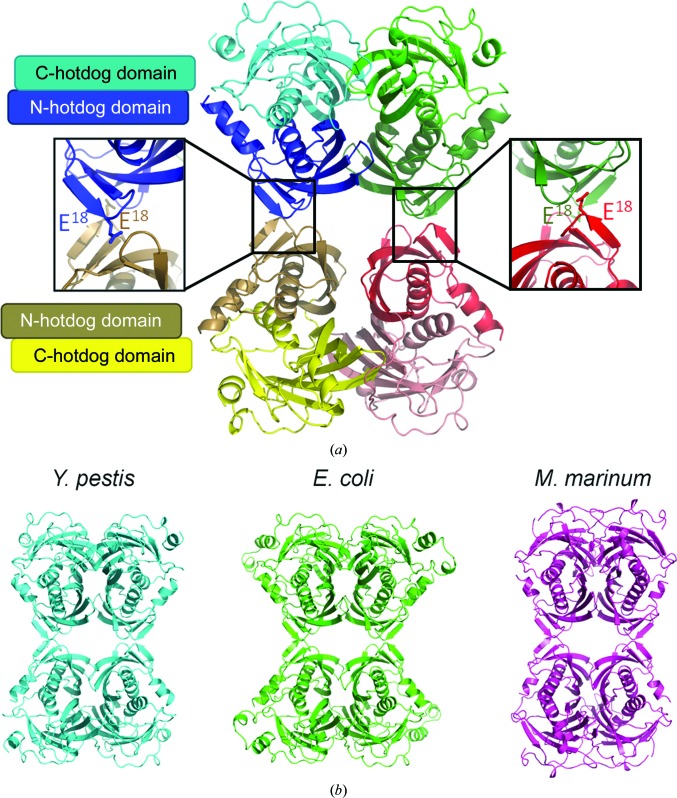
(*a*) The quaternary structure of TesB is a tetramer of protomers with a Glu residue mutated to disrupt the tetramer configuration (inset) which is conserved within the *E. coli* and *M. marinum* structures (*b*).

The quaternary state of TesB enzymes has not been well characterized in the literature. The asymmetric unit of our crystal structure contained four TesB double-hotdog protomers, and analysis of the binding interfaces suggested that either a dimer or a tetramer were possible functional quaternary structures Interestingly, an initial report on the crystallization of *Ec*TesB reported a homotetramer in the assymetric unit (Swenson *et al.*, 1994 ▶); however, the final structural determination reported a homodimer as the biological unit (Li *et al.*, 2000 ▶). We therefore set out to characterize the possible binding interfaces and quaternary structure. The strongest inter­actions were between a dimer of TesB protomers, with approximately 2500 Å^2^ of surface area at the interface, with the next strongest interactions between the two dimers within the asymmetric unit (approximately 890 Å^2^), forming an octamer of hotdog protomers (Fig. 4 ▶). To assess the biological state of the enzyme, we used a combination of biophysical techniques including analytical ultracentrifugation (AUC), size-exclusion chromatography (SEC) and small-angle X-ray scattering data.

**Figure 5:**
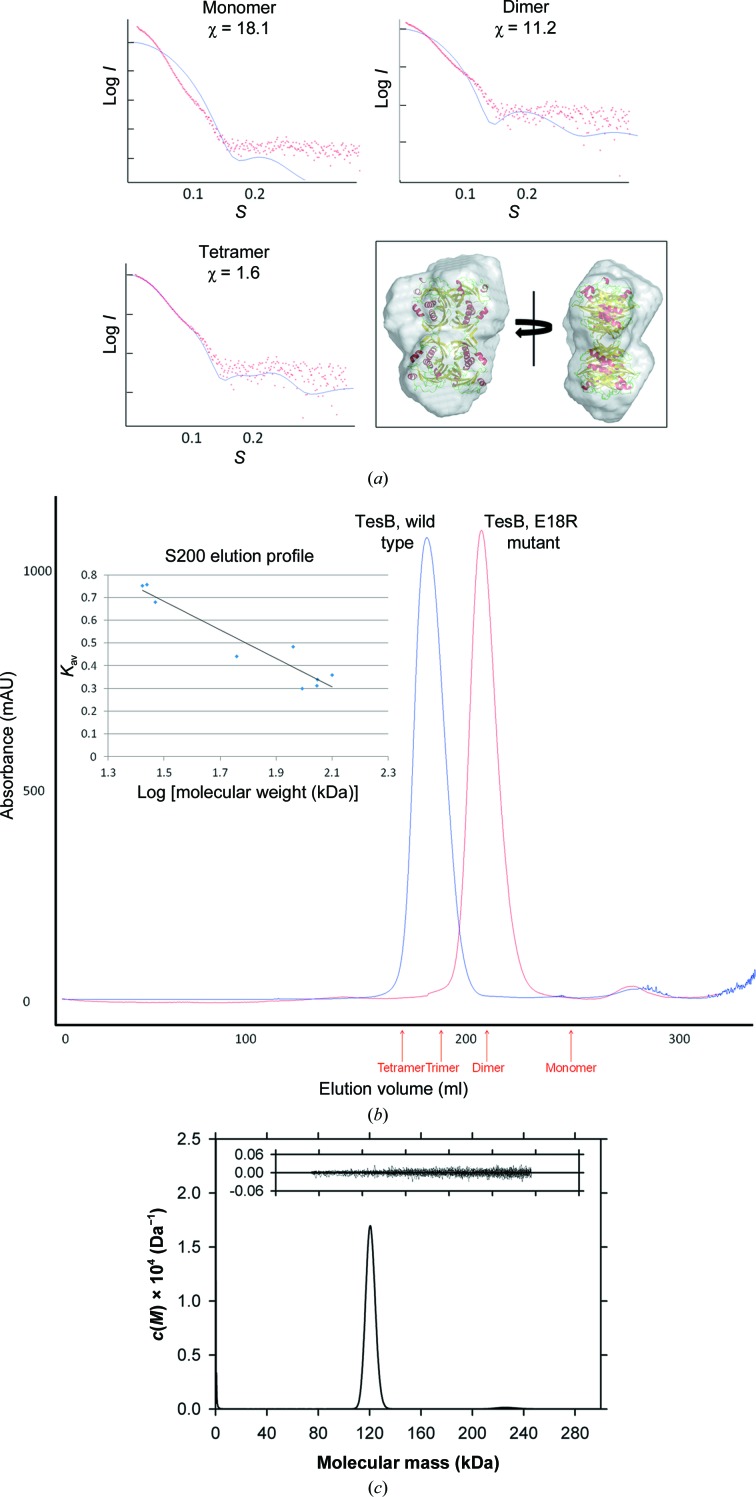
The tetramer was confirmed to be the biological unit using a number of biophysical techniques. (*a*) Small-angle X-ray scattering data were compared with scattering data generated using *CRYSOL* (Svergun *et al.*, 1995 ▶) for a monomer, a dimer and a tetramer, with the best fit for the tetramer, and a SAXS envelope was generated using the experimental data. (*b*) *Yp*TesB eluted from a size-exclusion column consistent with a tetramer, as confirmed using a standard curve of the size-exclusion column (inset) to determine the elution volumes of a monomer, a dimer, a trimer and a tetramer (red). (*c*) The continuous mass [*c*(*M*)] distribution is plotted as a function of molecular mass (kDa) for TesB (2.4 mg ml^−1^). The molecular mass at the ordinate maximum of the peak shown corresponds to 120 kDa. The *c*(*M*) distribution was calculated using 200 masses from 0 to 300 kDa at a *P*-value of 0.95, which resulted in an r.m.s.d. of 0.00685 and a runs test *Z* of 7.61 and yielded a frictional ratio of 1.28. Inset: residuals for the *c*(*M*) best fit plotted as a function of radial position.

Small-angle X-ray scattering data for TesB were collected over a concentration range of 0.3–5 mg ml^−1^ (Table 2 ▶). The radius of gyration (*R*_g_) calculated by Guinier analysis and with the pair-distribution function [*P*(*r*)] was determined to be 35.66 and 35.74 Å, respectively. The maximum dimension (*D*_max_) determined from the *P*(*r*) plot was 114 Å, which is consistent with an octameric configuration of thioesterase domains present in the asymmetric unit in the crystal structure (*D*_max_ = 114 Å). *CRYSOL* (Svergun *et al.*, 1995 ▶) was used to compare the different theor­etical scattering profiles of different possible multimeric states, ranging from monomer, dimer and tetramer configurations of the double-hotdog protomer, with the scattering data strongly suggesting a tetramer of double hotdogs present in the biological unit of the crystal (χ = 1.6), whilst a monomer and a dimer poorly fit the data (χ = 18.1 and χ = 11.2; Fig. 5 ▶ *a*). In addition, *DAMMIF* (Franke & Svergun, 2009 ▶) was also used to generate a dummy-atom model from the scattering data over 20 consecutive runs with a normalized spatial discrepancy of 0.871, with the tetrameric model showing the best qualitative fit to the shape of the *de novo* envelope (Fig. 5 ▶ *a*). Minor differences at the periphery between the crystal structure and the *DAMMIF*-derived SAXS model are possibly owing to flexible regions that could not be resolved in the crystal structure (for example, residues 139–154).

Consistent with these results, AUC demonstrated that *Yp*TesB exists as a single species in aqueous solution at an initial concentration of 2.4 mg ml^−1^ with a standardized sedimentation coefficient (*s*_20,w_) of 7.0 S and a molar mass of 120 kDa, consistent with the theoretical mass of a tetramer (130 kDa; Fig. 5 ▶ *c*). The SEC results were also consistent with TesB existing as a tetramer in solution, eluting from the column as a single peak at a volume consistent with that of a tetramer (Fig. 5 ▶ *b*).

Given the high structural similarity between the *Ec*TesB and *Yp*TesB monomers, we tested whether the same tetrameric structures could be generated in *Ec*TesB. Expanding the crystallographic symmetry in *Ec*TesB to generate different conformations revealed the same octameric arrangement as was observed in *Yp*TesB to also be present in the crystal structure of *Ec*TesB, and they contained similar interface areas, with a dimer interface 1 of 2500 Å^2^ and interface 2 of 860 Å^2^. Similarly, the structure of TesB from *M. marinum* deposited in the Protein Data Bank by a structural genomics consortium but as yet unpublished also contained the same arrangement, with one dimer interface of 2800 Å^2^ and the other of 860 Å^2^. Significantly, this octameric arrangement of hotdog dimers, confirmed in a range of biophysical assays and in two other crystal structures, has not been described in any other thioesterase and is likely to be a distinguishing feature of TesB-type thioesterases. To further confirm this quaternary structure, we introduced a mutation within a crucial region of the weaker biological interface that would disrupt the octamer state to a tetramer of hotdog domains (see Fig. 4 ▶ *a*). Recombinant expression and purification of the Glu18-to-Arg18 mutation confirmed that the oligomeric state of the enzyme was clearly disrupted to the expected tetrameric state (see Fig. 5 ▶ *b*).

### 3.4. TesB exhibits specificity for octanoyl-CoA   

**Figure 6:**
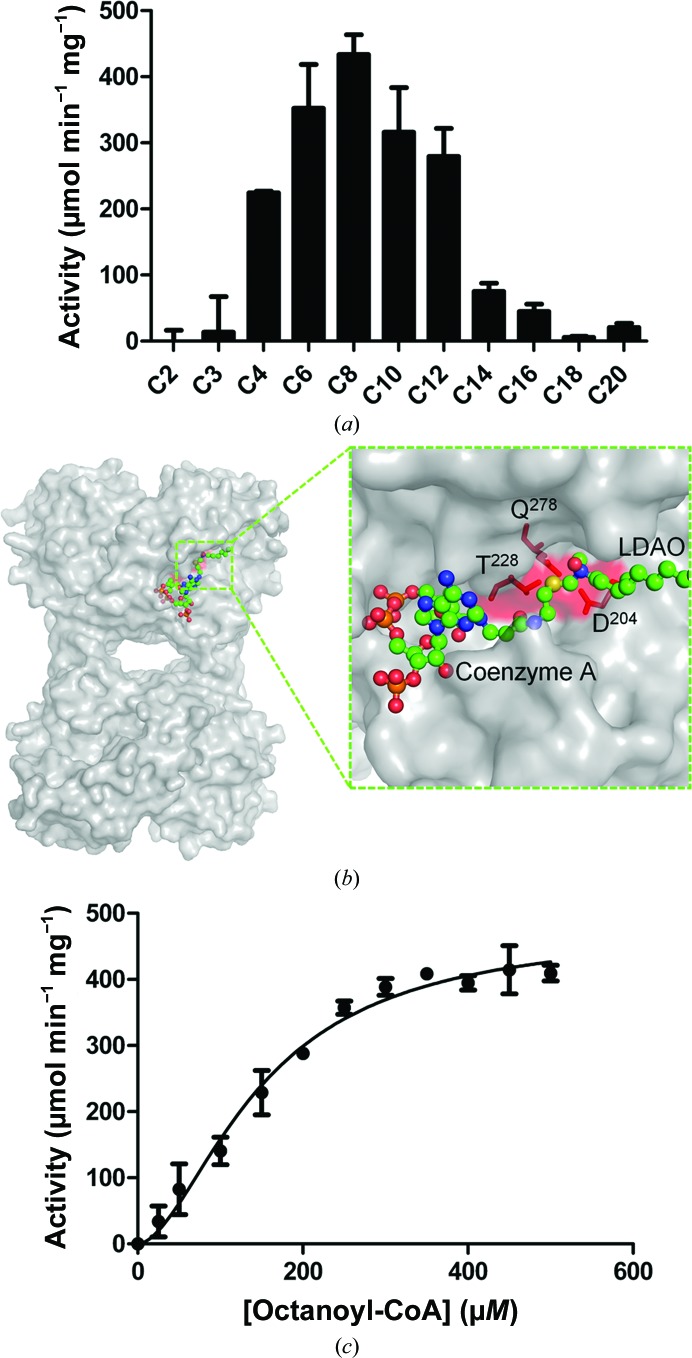
TesB activity against a range of substrates (*a*), demonstrating activity against a broad range of fatty acyl-CoA chain lengths; octanoyl-CoA was identified as the preferred substrate and was investigated further. (*b*) The active site with CoA and LDAO (from the *Ec*TesB model) superimposed. (*c*) Activity curve for octanoyl-CoA, with a Hill coefficient of 1.753 and a *V*_max_ of 478 µmol min^−1^ mg^−1^.

Since neither the substrate nor the biological role of *Yp*TesB has been determined, we set out to test the activity of a range of acyl-CoA substrates. *Yp*TesB activity for substrates ranging from short-chain (C2, C3, C4) and medium-chain (C6, C8, C10, C12) to long-chain (C14, C16, C18, C20) fatty acyl-CoA substrates were tested using an established enzyme-activity assay (Hunt *et al.*, 2002 ▶; Yamada *et al.*, 1996 ▶). The highest activity was exhibited towards medium-chain acyl-CoAs (C6–C10), with a peak of activity observed for C8 (Fig. 6 ▶ *a*).

The activity profile is similar to the reported specificity for the human homologue ACOT8 by Watanabe *et al.* (1997 ▶), which showed a preference for medium-chain (C4–C10) acyl-CoAs. The activity profile for octanoyl-CoA revealed a sigmoidal relationship, with a Hill coefficient of 1.75 (Fig. 6*c* ▶), suggestive of positive regulation between the protomers; this is consistent with the activity profiles of both human ACOT8 and the plant homologue acyl-CoA hydrolase 2 (ACH2), which also exhibit similar sigmoidal activity profiles suggestive of substrate cooperativity (Tilton *et al.*, 2004 ▶; Watanabe *et al.*, 1997 ▶; see also the CoA-bound structure at half of sites discussed below). The specificity of *Yp*TesB for medium-chain fatty acyl-CoAs, in combination with the fact that TesB is upregulated during β-oxidation (Tilton *et al.*, 2004 ▶), provides further support for a role of TesB in *Y. pestis* in the removal of products of β-oxidation at specific chain lengths and/or in potentially preventing the sequestration of CoASH into activated fatty acids from limiting the flow of short-chain fatty acids into β-oxidation (Tilton *et al.*, 2004 ▶).

### 3.5. The CoA-bound structure of TesB reveals the active-site pocket   

**Figure 7:**
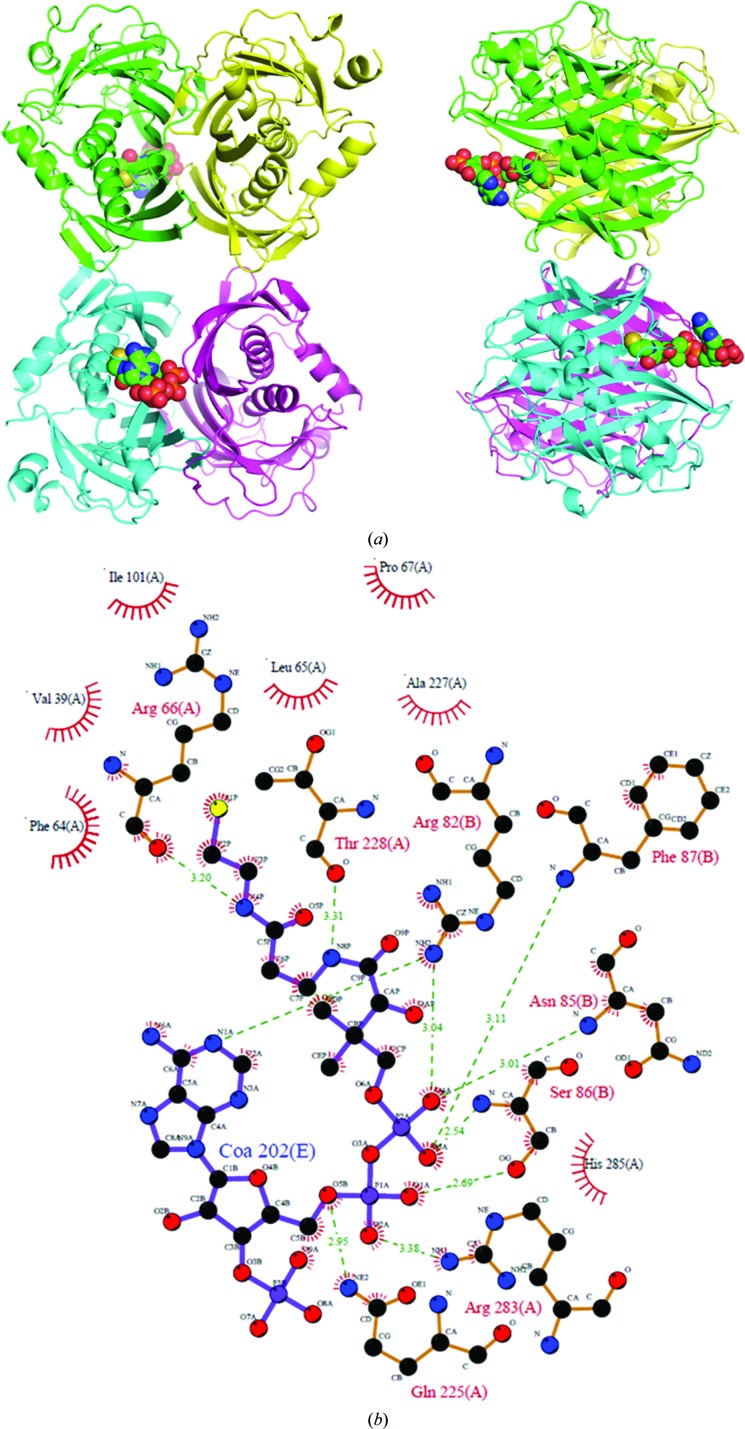
(*a*) The structure of TesB solved in the presence of CoA; the same octameric configuration is observed as for the apo form of the enzyme. (*b*) *LigPlot* representation of the detailed interactions of CoA with TesB (Wallace *et al.*, 1995 ▶).

Since the previously determined *Ec*TesB structure contained a lipid molecule of similar chain length to C8, we set out to determine the CoA binding site of TesB enzymes to assess whether the lipid-bound moiety could be a basis for modelling the octanoyl-CoA binding site. Crystals of CoA-bound *Yp*TesB diffracted to 2.2 Å resolution, revealing the same quaternary arrangement of domains as the apo form with density for two CoA molecules. CoA was wedged between two adjacent chains and binding was mediated through interactions with Arg66, Thr228, Arg283 and Gln225 of one chain and Arg82, Phe87, Asn85 and Ser86 of the adjacent chain (Fig. 7 ▶ *b*). CoA binding also provided additional density for the flexible loop regions that are missing in the apo *Yp*TesB structure. Superposition of the *Yp*TesB–CoA structure with the *Ec*TesB–LDAO structure revealed that the terminal S atom of CoA, which is responsible for forming the thioester bond in fatty-acyl substrates, is in close proximity (5 Å) to the LDAO (Fig. 6 ▶ *b*). That these binding regions are likely to represent the binding domains of octanoyl-CoA is further supported by the close proximity of the conserved active-site residues Asp204, Thr228 and Gln278 (Fig. 3 ▶). Interestingly, only two of a possible four identical CoA binding sites contained CoA. Superposition of CoA into the unbound sites did not reveal any major clashes nor crystallo­graphic packing perturbations, and thus whilst neither a structural or functional basis for this half-of-sites binding is clear, this half-of-sites activity has been noted across a wide range of thio­esterase structures published to date (Forwood *et al.*, 2007 ▶; Marfori *et al.*, 2011 ▶; Swarbrick *et al.*, 2014 ▶) and is present in other structures (*e.g* PDB entries 2qq2 and 4moc; Structural Genomics Consortium, unpublished work; Swarbrick *et al.*, 2014 ▶). The presence of two thioesterase domains is also observed in the TE6 family, and is possibly the result of a gene-duplication and fusion event, since both individual domains possessed similar monomer and quaternary arrangements. The activity of the individual domains has previously been investigated by Forwood *et al.* (2007 ▶), demonstrating that each thioesterase domain expressed individually resulted in two inactive domains which, when combined, were able to rescue the activity, suggesting that both domains were required for activity, with mutagenesis and structural analysis confirming a half-of-sites activity.

## 4. Conclusion   

Here, we present the first structural and functional characterization of TesB from *Y. pestis*, providing new insights into the TE4 thioesterase family. These structural features are conserved within TesB structures, thus representing distinguishing features for this enzyme class. The structure of the protomer exhibits two face-to-face hotdog domains connected through a long 23-residue linker. Interestingly, this double-hotdog protomer in *Yp*TesB associates into a tetramer both in the crystal and in solution as determined across a range of biophysical assays. This octameric arrangement of hotdog domains (a tetramer of double-hotdog protomers) is not present in any other thioesterase family and has not been described in the literature, and thus represents a new quaternary arrangement in this superfamily. That the same configuration is likely to be present in the two other TesB structures solved to date strongly suggests that this arrangement is likely to be a conserved feature of TesB thioesterases. Other distinguishing features include a π-helix that spans the active site and which interrupts the central α-helix within the second hotdog domain, the lack of a C-terminal α-helix that is common among other hotdog-domain thioesterase families and the conserved active-site residues Asp204, Thr228 and Gln278. The structure also provides a basis for the observed specificity for octanoyl-CoA and other medium-chain fatty-acyl CoAs.

## Supplementary Material

PDB reference: TesB, 4qfw

PDB reference: complex with coenzyme A, 4r4u

Supporting Information.. DOI: 10.1107/S1399004715002527/mn5084sup1.pdf

## Figures and Tables

**Table 1:** Data-collection, processing and refinement statistics for TesB Values in parentheses are for the highest resolution shell.

	*Yp*TesB (PDB entry 4qfw)	*Yp*TesB + CoA (PDB entry 4r4u)
Wavelength ()	0.9537	0.9537
Resolution range ()	36.892.00 (2.042.00)	45.912.20 (2.262.20)
Space group	*P*12_1_1	*P*12_1_1
Unit-cell parameters (, )	*a* = 51.23, *b* = 171.62, *c* = 73.70, = 90, = 109.62, = 90	*a* = 550.98, *b* = 171.37, *c* = 73.66, = 90, = 109.51, = 90
Total observations	446767 (25650)	461970 (33455)
Unique reflections	75550 (4191)	60243 (4431)
Multiplicity	5.9 (6.1)	7.7 (7.6)
Completeness (%)	93.9 (87.3)	100 (100)
*R*_merge_	0.103 (0.211)	0.105 (0.491)
*R*_p.i.m._	0.047 (0.09)	0.043 (0.206)
Mean *I*/(*I*)	10.3 (5.6)	10.3 (4.1)
Wilson *B* value (^2^)	28.85	32.44
*R*_cryst_	0.22	0.20
*R*_free_	0.27	0.26
No. of atoms		
Total	8667	9126
Macromolecules	8460	8712
Water	207	414
R.m.s.d., bonds ()	0.017	0.009
R.m.s.d., angles ()	1.613	1.152
Ramachandran plot (%)		
Favoured region	91.8	90.4
Allowed region	7.5	9.4
Generously allowed region	0.7	0.2
Disallowed region	0.0	0.0

**Table 2:** Data-collection and scattering-derived parameters for TesB SAXS data

Data-collection parameters	
Instrument	Pilatus 1M
Beam geometry	250 150m
Wavelength ()	1.54
*q* range (^1^)	0.0090.541
Exposure time (s)	21.0
Concentration range (mgml^1^)	0.35
Temperature (C)	16
Structural parameters	
*I*(0) (cm^1^) [from *P*(*r*)]	0.05
*R*_g_ () [from *P*(*r*)]	35.74
*I*(0) (cm^1^) (from Guinier)	0.05
*R*_g_ () (from Guinier)	35.66 0.45
*D*_max_ ()	114
Porod volume estimate (^3^)	156683
Dry volume calculated from sequence (^3^)	39278
Molecular-mass determination	
Partial specific volume (cm^3^g^1^)	0.75
Contrast ( 10^10^ cm^2^)	2.67
Molecular mass *M*_r_ (Da)	125145
Calculated monomeric *M*_r_ from sequence (Da)	32462

## References

[bb1] Cabanel, N., Leclercq, A., Chenal-Francisque, V., Annajar, B., Rajerison, M., Bekkhoucha, S., Bertherat, E. & Carniel, E. (2013). *Emerg. Infect. Dis.* **19**, 230–236. 10.3201/eid1902.121031 PMC3559055 23347743

[bb2] Cantu, D. C., Chen, Y. & Reilly, P. J. (2010). *Protein Sci.* **19**, 1281–1295. 10.1002/pro.417 PMC2974821 20506386

[bb3] Cooley, R. B., Arp, D. J. & Karplus, P. A. (2010). *J. Mol. Biol.* **404**, 232–246. 10.1016/j.jmb.2010.09.034 PMC2981643 20888342

[bb4] Dias, M. V., Huang, F., Chirgadze, D. Y., Tosin, M., Spiteller, D., Dry, E. F., Leadlay, P. F., Spencer, J. B. & Blundell, T. L. (2010). *J. Biol. Chem.* **285**, 22495–22504. 10.1074/jbc.M110.107177 PMC2903362 20430898

[bb5] Dillon, S. C. & Bateman, A. (2004). *BMC Bioinformatics*, **5**, 109. 10.1186/1471-2105-5-109 PMC516016 15307895

[bb6] Emsley, P., Lohkamp, B., Scott, W. G. & Cowtan, K. (2010). *Acta Cryst.* D**66**, 486–501. 10.1107/S0907444910007493 PMC2852313 20383002

[bb7] Evans, P. R. (2011). *Acta Cryst.* D**67**, 282–292. 10.1107/S090744491003982X PMC3069743 21460446

[bb8] Forwood, J. K., Thakur, A. S., Guncar, G., Marfori, M., Mouradov, D., Meng, W., Robinson, J., Huber, T., Kellie, S., Martin, J. L., Hume, D. A. & Kobe, B. (2007). *Proc. Natl Acad. Sci. USA*, **104**, 10382–10387. 10.1073/pnas.0700974104 PMC1965522 17563367

[bb9] Franke, D. & Svergun, D. I. (2009). *J. Appl. Cryst.* **42**, 342–346. 10.1107/S0021889809000338 PMC5023043 27630371

[bb10] Galimand, M., Carniel, E. & Courvalin, P. (2006). *Antimicrob. Agents Chemother.* **50**, 3233–3236. 10.1128/AAC.00306-06 PMC1610074 17005799

[bb11] Holm, L. & Rosenström, P. (2010). *Nucleic Acids Res.* **38**, W545–W549. 10.1093/nar/gkq366 PMC2896194 20457744

[bb12] Hunt, M. C., Solaas, K., Kase, B. F. & Alexson, S. E. (2002). *J. Biol. Chem.* **277**, 1128–1138. 10.1074/jbc.M106458200 11673457

[bb13] Inglesby, T. V. *et al.* (2000). *JAMA*, **283**, 2281–2290. 10.1001/jama.283.17.2281 10807389

[bb14] Kirkby, B., Roman, N., Kobe, B., Kellie, S. & Forwood, J. K. (2010). *Prog. Lipid Res.* **49**, 366–377. 10.1016/j.plipres.2010.04.001 20470824

[bb15] Konarev, P. V., Volkov, V. V., Sokolova, A. V., Koch, M. H. J. & Svergun, D. I. (2003). *J. Appl. Cryst.* **36**, 1277–1282.

[bb16] Kool, J. L. (2005). *Clin. Infect. Dis.* **40**, 1166–1172. 10.1086/428617 15791518

[bb17] Laue, T. M., Shah, B. D., Ridgeway, T. M. & Pelletier, S. L. (1992). *Analytical Ultracentrifugation in Biochemistry and Polymer Science*, pp. 90–125. Cambridge: The Royal Society of Chemistry.

[bb18] Leslie, A. G. W. & Powell, H. R. (2007). *Evolving Methods for Macromolecular Crystallography*, edited by R. J. Read & J. L. Sussman, pp. 41–51. Dordrecht: Springer.

[bb19] Li, J., Derewenda, U., Dauter, Z., Smith, S. & Derewenda, Z. S. (2000). *Nature Struct. Mol. Biol.* **7**, 555–559. 10.1038/76776 10876240

[bb20] Marfori, M., Kobe, B. & Forwood, J. K. (2011). *J. Biol. Chem.* **286**, 35643–35649. 10.1074/jbc.M111.225953 PMC3195577 21849495

[bb21] McCoy, A. J., Grosse-Kunstleve, R. W., Adams, P. D., Winn, M. D., Storoni, L. C. & Read, R. J. (2007). *J. Appl. Cryst.* **40**, 658–674. 10.1107/S0021889807021206 PMC2483472 19461840

[bb22] McNicholas, S., Potterton, E., Wilson, K. S. & Noble, M. E. M. (2011). *Acta Cryst.* D**67**, 386–394. 10.1107/S0907444911007281 PMC3069754 21460457

[bb23] Murshudov, G. N., Skubák, P., Lebedev, A. A., Pannu, N. S., Steiner, R. A., Nicholls, R. A., Winn, M. D., Long, F. & Vagin, A. A. (2011). *Acta Cryst.* D**67**, 355–367. 10.1107/S0907444911001314 PMC3069751 21460454

[bb24] Perugini, M. A., Schuck, P. & Howlett, G. J. (2000). *J. Biol. Chem.* **275**, 36758–36765. 10.1074/jbc.M005565200 10970893

[bb25] Pidugu, L. S., Maity, K., Ramaswamy, K., Surolia, N. & Suguna, K. (2009). *BMC Struct. Biol.* **9**, 37–53. 10.1186/1472-6807-9-37 PMC2698920 19473548

[bb26] Schuck, P. (2000). *Biophys. J.* **78**, 1606–1619. 10.1016/S0006-3495(00)76713-0 PMC1300758 10692345

[bb27] Schuck, P., Perugini, M. A., Gonzales, N. R., Howlett, G. J. & Schubert, D. (2002). *Biophys. J.* **82**, 1096–1111. 10.1016/S0006-3495(02)75469-6 PMC1301916 11806949

[bb28] Svergun, D., Barberato, C. & Koch, M. H. J. (1995). *J. Appl. Cryst.* **28**, 768–773.

[bb29] Swarbrick, C. M. D., Patterson, E. I. & Forwood, J. K. (2013). *Acta Cryst.* F**69**, 188–190. 10.1107/S1744309113001267 PMC3564626 23385765

[bb30] Swarbrick, C. M. D., Roman, N., Cowieson, N., Patterson, E. I., Nanson, J., Siponen, M. I., Berglund, H., Lehtiö, L. & Forwood, J. K. (2014). *J. Biol. Chem.* **289**, 24263–24274. 10.1074/jbc.M114.589408 PMC4148856 25002576

[bb31] Swarbrick, C. M. D., Roman, N. & Forwood, J. K. (2011). *Inflammatory Diseases – A Modern Perspective*, edited by A. Nagal, pp. 203–218. Rijeka: InTech.

[bb32] Swenson, L., Green, R., Smith, S. & Derewenda, Z. S. (1994). *J. Mol. Biol.* **236**, 660–662. 10.1006/jmbi.1994.1175 8107148

[bb33] Tilton, G. B., Shockey, J. M. & Browse, J. (2004). *J. Biol. Chem.* **279**, 7487–7494. 10.1074/jbc.M309532200 14660652

[bb34] Tourdjman, M., Ibraheem, M., Brett, M., DeBess, E., Progulske, B., Ettestad, P., McGivern, T., Petersen, J. & Mead, P. (2012). *Clin. Infect. Dis.* **55**, e58–e60. 10.1093/cid/cis578 22715170

[bb35] Wallace, A. C., Laskowski, R. A. & Thornton, J. M. (1995). *Protein Eng. Des. Sel.* **8**, 127–134. 10.1093/protein/8.2.127 7630882

[bb36] Watanabe, H., Shiratori, T., Shoji, H., Miyatake, S., Okazaki, Y., Ikuta, K., Sato, T. & Saito, T. (1997). *Biochem. Biophys. Res. Commun.* **238**, 234–239. 10.1006/bbrc.1997.7217 9299485

[bb37] Willis, M. A., Zhuang, Z., Song, F., Howard, A., Dunaway-Mariano, D. & Herzberg, O. (2008). *Biochemistry*, **47**, 2797–2805. 10.1021/bi702336d 18260643

[bb38] Yamada, J., Furihata, T., Tamura, H., Watanabe, T. & Suga, T. (1996). *Arch. Biochem. Biophys.* **326**, 106–114. 10.1006/abbi.1996.0053 8579357

[bb39] Yamada, J., Kurata, A., Hirata, M., Taniguchi, T., Takama, H., Furihata, T., Shiratori, K., Iida, N., Takagi-Sakuma, M., Watanabe, T., Kurosaki, K., Endo, T. & Suga, T. (1999). *J. Biochem.* **126**, 1013–1019. 10.1093/oxfordjournals.jbchem.a022544 10578051

