# Supplementary material for: Structural and functional characterization of TesB from Yersinia pestis reveals a unique octameric arrangement of hotdog domains
Source: Acta Crystallogr D Biol Crystallogr. 2015 Mar 27;71(Pt 4):986–95. doi: 10.1107/S1399004715002527 (PMC4388271; doi:10.1107/S1399004715002527)
Supplement: Supplementary file 1 [file d-71-00986-sup1.pdf]

Acta Cryst D BIOLOGICAL CRYSTALLOGRAPHY

Volume 71 (2015)

Supporting information for article:

Structural and functional characterization of TesB from *Yersinia pestis* reveals a unique octameric arrangement of hotdog domains

C. M. D. Swarbrick, M. E. Perugini, N. Cowieson and J. K. Forwood

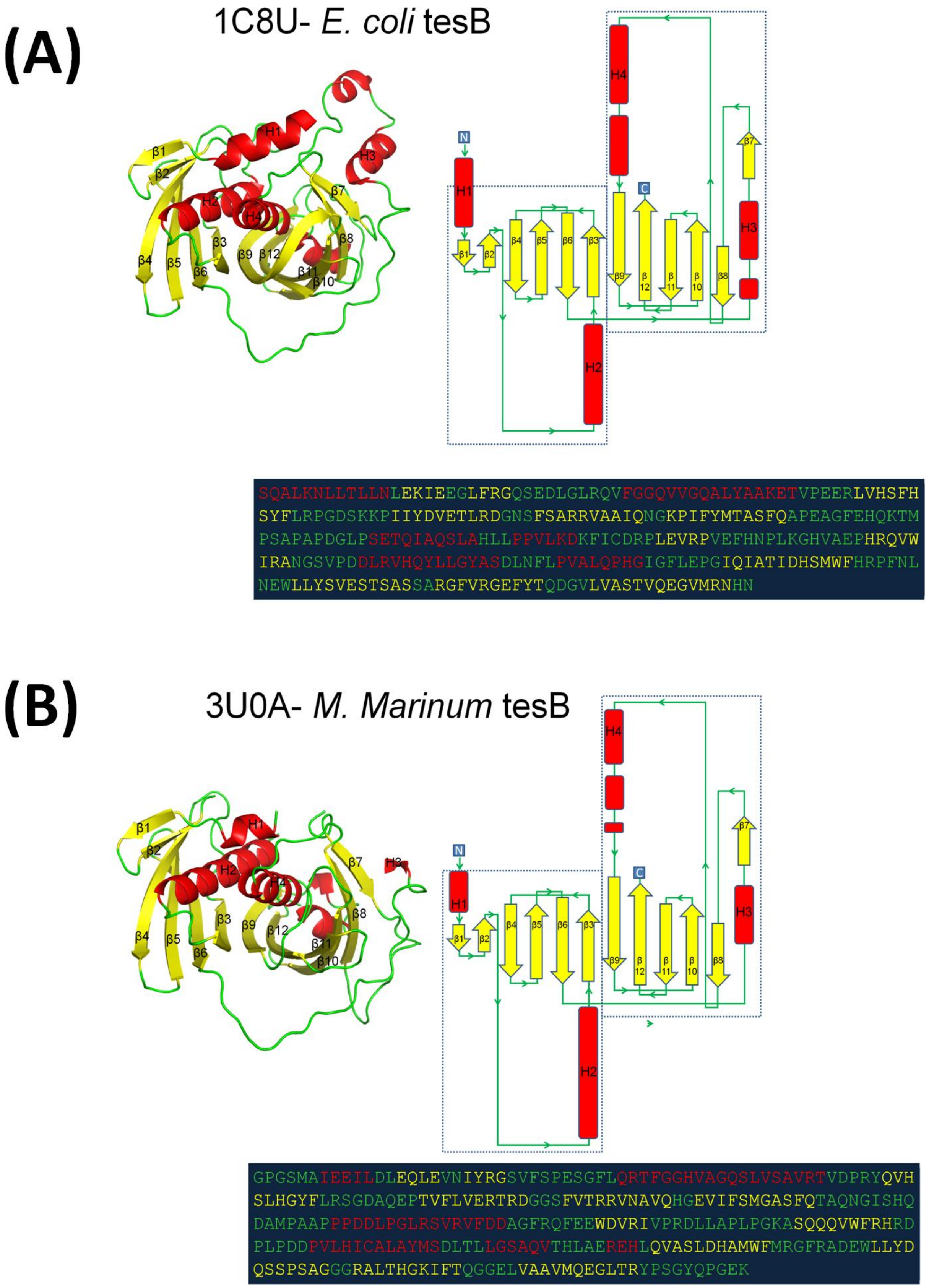

Figure S1 The primary, secondary and tertiary structure of (A) *Ec*tesB and (B) *Mm*tesB with α-helices coloured red, β-stands in yellow, and loops in green.

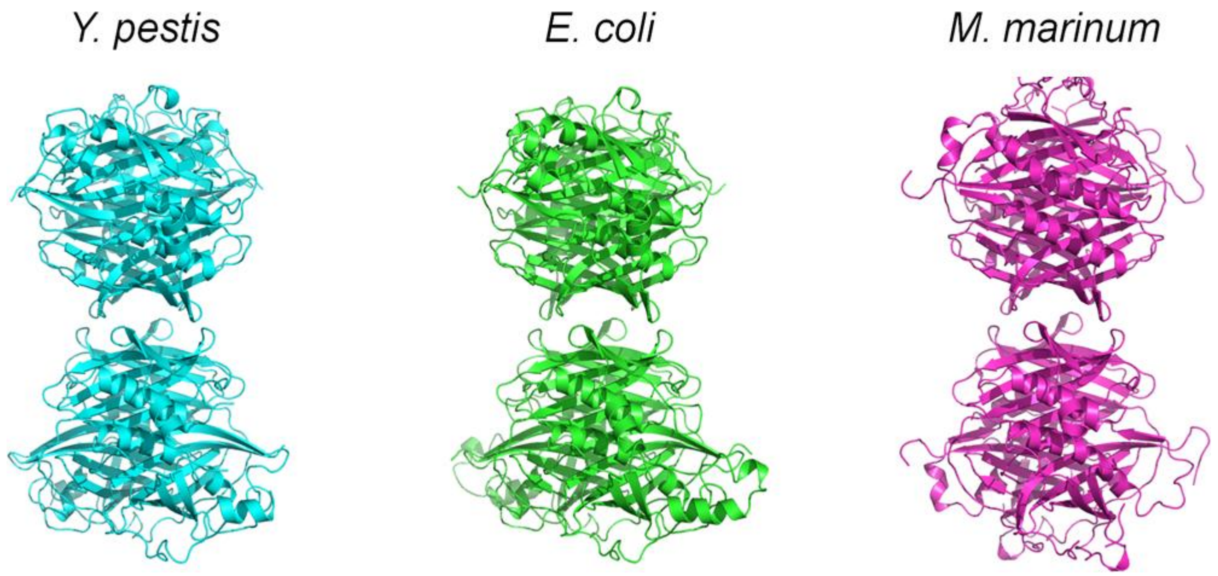

Figure S2 Alternate view of the *Y. pestis*, *E. coli* and *M. marinum* structures.

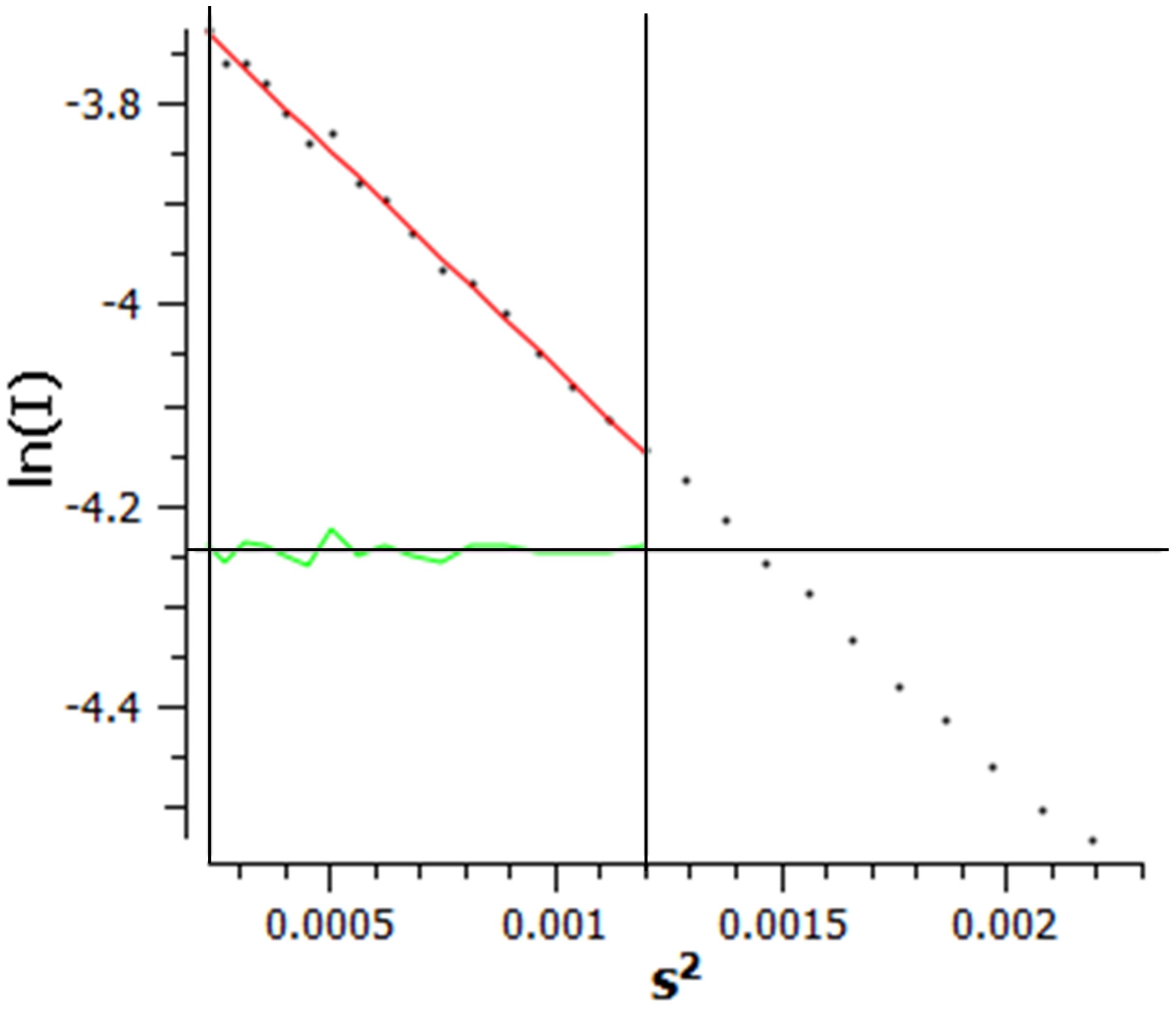

Figure S3 Guinier plot of ln(I) vs S^2 demonstrating the protein sample from which SAXS data was derived is free of aggregation.

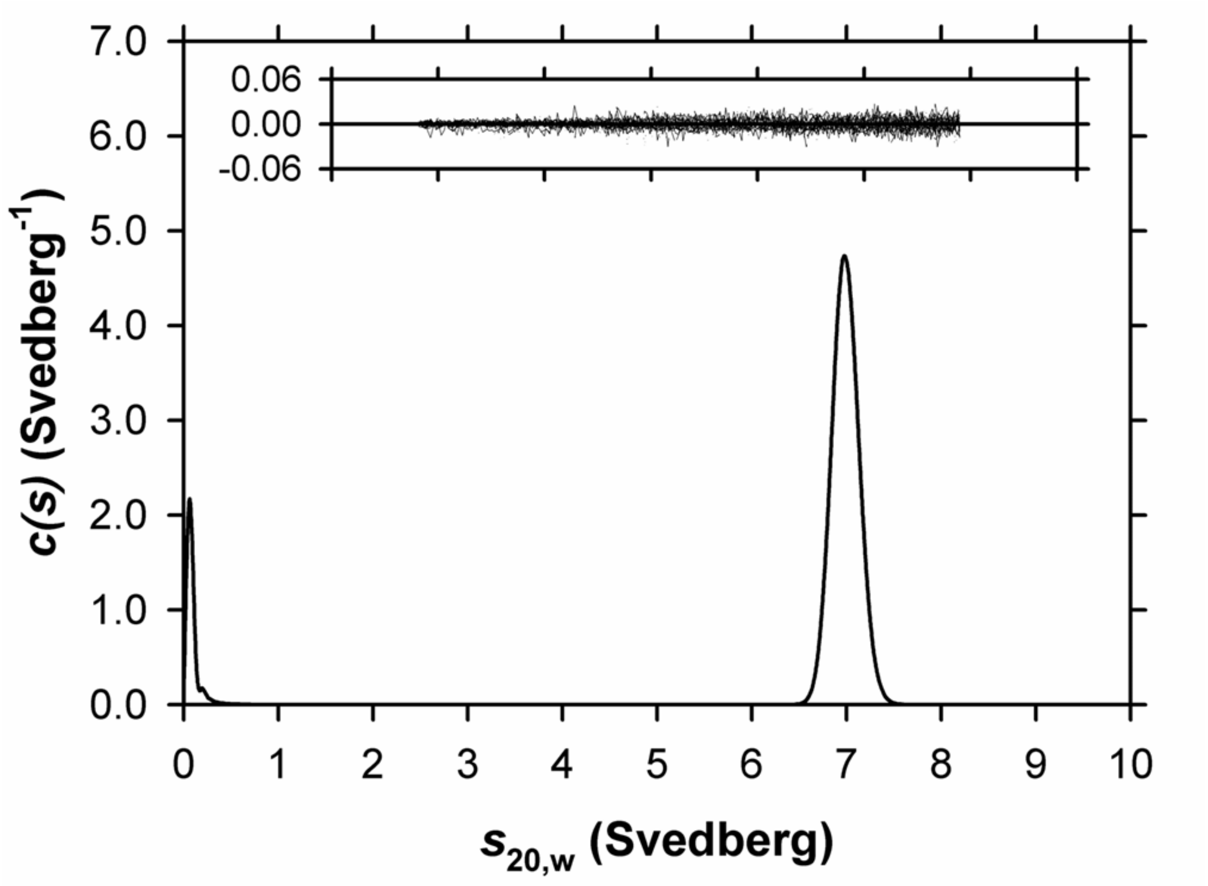

Figure S4 Sedimentation velocity analysis of TesB. The continuous sedimentation coefficient [c(s)] distribution is plotted as a function of standardized sedimentation (Svedberg) for TesB (2.4 mg/ml). The sedimentation coefficient at the ordinate maximum of the peak shown corresponds to 7.0 S. The c(s) distribution was calculated using 200 sedimentation coefficients from 0 to 10 S at a P-value of 0.95, which resulted in a RMSD of 0.00686, Runs test Z = 7.39 and yielded a frictional ratio of 1.28. Inset - Residuals for the c(s) best fit plotted as a function of radial position.
